# Lysozyme supplement enhances antioxidant capacity and regulates liver glucolipid metabolism in weaned piglets

**DOI:** 10.3389/fvets.2025.1721300

**Published:** 2025-12-03

**Authors:** Yuying Wu, Huihui Zhang, Yonggang Zhang, Chunyan Xie, Guoshun Chen

**Affiliations:** 1 College of Animal Science and Technology, Gansu Agricultural University, Lanzhou, China; 2 Tianjin Key Laboratory of Agricultural Animal Breeding and Healthy Husbandry, College of Animal Science and Veterinary Medicine, Tianjin Agricultural University, Tianjin, China; 3 Huanshan Group Co., Ltd, Qingdao, China; 4 Tianjin Key Laboratory of Animal Molecular Breeding and Biotechnology, Tianjin Livestock and Poultry Health Breeding Technology Engineering Center, Institute of Animal Science and Veterinary, Tianjin Academy of Agricultural Sciences, Tianjin, China

**Keywords:** lysozyme (LZ), weaned piglet, lipid metabolism, glucose metabolism, antioxidant function

## Abstract

**Introduction:**

Lysozyme (LZ), a naturally occurring antimicrobial peptide, has demonstrated beneficial bioactivities. This study aimed to investigate the effects of dietary LZ supplementation on hepatic antioxidant function and glucolipid metabolism in weaned piglets.

**Methods:**

Forty-eight weaned piglets (Landrace × Yorkshire, 22 days old) were randomly assigned to two dietary treatments: a control (CON) group fed a basal diet, and an LZ group fed the basal diet supplemented with 0.1% LZ for 19 days. Liver index and serum biochemical parameters were measured. Hepatic antioxidant enzyme activities and the mRNA expression of genes related to antioxidant function, lipid metabolism, and gluconeogenesis were determined. Liver fatty acid profiles were also analyzed.

**Results:**

Dietary LZ supplementation significantly increased the liver index and serum concentrations of triglyceride (TG), total cholesterol (TC), and Low-Density Lipoprotein Cholesterol (LDLC) (*p* < 0.05). In the liver, LZ significantly enhanced catalase (*CAT*) activity and up-regulated the mRNA expression of antioxidant genes, including *NQO1*, *Nrf2*, *MnSOD*, and *CAT* (*p* < 0.05). Furthermore, LZ altered the hepatic fatty acid profile by increasing the contents of C16:1, C17:0, C20:3n6, and C18:1n9t (*p* < 0.05). Gene expression analysis revealed that LZ up-regulated *CPT1α* and *PPARα* (*p* < 0.05) but down-regulated *SCD* and *SREBP1* (*p* < 0.05). Additionally, LZ supplementation significantly increased the mRNA expression of gluconeogenic enzymes, including PEPCK, PC, and G6PC (*p* < 0.05).

**Discussion:**

The results indicate that dietary LZ supplementation enhances the hepatic antioxidant defense system, likely through activation of the Nrf2 signaling pathway. Concurrently, LZ modulates lipid metabolism by promoting fatty acid oxidation (via up-regulation of *PPARα* and *CPT1α*) and inhibiting lipogenesis (via down-regulation of *SREBP1* and *SCD*). The up-regulation of key gluconeogenic genes suggests improved hepatic glucose production. In conclusion, LZ improves antioxidant capacity and regulates glucolipid metabolism in the liver of weaned piglets, supporting its potential as a functional feed additive in pig production.

## 1 Introduction

In modern pig husbandry systems, weaning is a critical period for pig feeding (1, 2). However, weaning often results in stress, thereby reducing nutrient digestion and absorption (3). Piglets are born with limited energy stores and a restricted capacity to oxidize fatty acids and amino acids; thus, insufficient energy supply may ultimately cause death in weaned piglets (4). The liver plays a crucial role in energy utilization and maintaining normal hepatic energy metabolism (5). Dietary intervention provides a beneficial approach for meeting the nutritional needs of weaned piglets while supporting their health. Following China’s complete prohibition of antibiotic growth promoters in animal feed in 2020, developing novel, safe, and effective feed additives has become an important research priority for sustainable pig production (6). Consequently, there is an urgent need to identify a natural and effective feed additive to alleviate oxidative stress in piglets.

Lysozyme (LZ) is a protein peptide with natural antibacterial activity, widely distributed in animal and plant tissues as well as their secretions (7, 8). Accumulating evidence indicates that LZ exhibits beneficial bioactivities, including anti-inflammatory, anti-obesity, and intestinal microbiota-modulating effects (9). Significantly, LZ has demonstrated effective antioxidant capability. An *in vivo* study evaluating the impact of dietary LZ on antioxidant capacity in juvenile gibel carp reported a significant increase in serum superoxide dismutase (SOD) activity and a marked decrease in malondialdehyde content in the mid-intestine (10). Furthermore, LZ supplementation in broiler chickens induced upregulation of intestinal SOD1 and GSH-Px (11). However, the effects of LZ supplementation on hepatic glucose and lipid metabolism have not yet been studied.

This study aimed to evaluate the effects of dietary LZ supplementation on hepatic glucose and lipid metabolism, as well as antioxidant function in weaned piglets. The findings from this research will provide important insights into the molecular mechanisms by which LZ modulates metabolic pathways, supporting its potential application as a functional feed additive in swine production.

## 2 Materials and methods

### 2.1 Animal ethics statement

The experimental design and procedure used in this study were approved by the Animal Care and Use Committee of the Institute of Subtropical Agroecology, Chinese Academy of Sciences (IACUC # 201302).

### 2.2 Experimental materials

The LZ used in the experiment was provided by Zhumadian Huazhong Chia Tai Co. Ltd. (Zhumadian, China) in powdered form with an activity of 50 U/mg (0.1%) and the main effective component being LZ dimer (12). The basal diet (Table 1) met or exceeded the nutritional requirements of weaned piglets as recommended by the United States National Research Council 1998.

**Table 1:** Ingredients and nutrient composition of the piglet diets (as-fed basis, %).

Item	Content
Ingredients	
Corn	38.35
Rice (broken)	10.00
Wheat flour	12.80
Soy protein concentrate	4.00
Soybean meal (CP, 46%)	13.48
Soy oil	1.50
Fish meal (CP, 65%)	4.00
Full-fat soybean	4.00
Whey power (CP, 3.8%)	3.76
Glucose	2.00
CaHPO_4_	0.83
Calcium lactate	0.85
Salt	0.14
Sugar	2.00
L-Lysine·HCl (78.8%)	0.39
DL-Methionine (99%)	0.08
L-Threonine (98.5%)	0.12
Lycine	0.10
Choline (60%)	0.07
Nucleotides	1.00
Flavoring agent	0.13
Vitamin-mineral premix ^1^	0.4
Total	100
Calculated values of nutrient levels	
Crude protein	19.37
Digestible energy, MJ/kg	15.36
Crude fat	4.52
Crude fibre	1.92
Crude ash	3.89
Salt	0.24
Ca	0.72
Available P	0.45
Lys	1.60
Met	0.67
Cys	0.47
Thr	1.07
Tyr	0.30

^1^Premix provided the following per kilogram of diet: Fe (FeSO_4_·H_2_O), 80 mg; Mn (MnSO_4_·5H_2_O), 45 mg; Zn (ZnO), 100 mg; Cu (CuSO_4_·5H_2_O), 20 mg; I (KI), 0.70 mg; Se (Na_2_SeO_3_·H_2_O), 0.25 mg; vitamin A, 10,000 IU; vitamin D_3_, 2,500 IU; vitamin E, 100 IU; vitamin K, 10 IU; vitamin B_2_, 7 mg; vitamin B_6_, 3 mg; vitamin B_12_ 50 μg; biotin, 80 μg; folic acid, 0.5 mg; nicotinic acid, 15 mg; choline chloride 1,500 mg.

### 2.3 Experimental design and treatments

In this experiment, 48 healthy weaned piglets (Landrace × Yorkshire, half male and half female) aged 22 d and of similar size were selected from 6 litters, and were randomly divided into 2 groups with 6 replicates in each group and 4 piglets in each replicate. Dietary treatments were designed as follows: (1) control group (CON, basal diet); (2) LZ group (LZ, basal diet + 0.1% LZ), and the experiment lasted for 19 d. Piglets in each replicate were bred in 1 pen, the ambient temperature was controlled at about 28 °C to 24 °C and the humidity was controlled at 65 to 75%. Piglets were fed 6 times a day (at 06:30, 09:00, 11:30, 14:30, 17:30 and 22:00, respectively) and had free access to water. All piglets were vaccinated according to the regulations of the farm. Throughout the experimental period, all piglets remained healthy, and no mortality or morbidity occurred.

### 2.4 Sample collection

On the final day of the trial, 6 pigs were randomly selected from each treatment for blood and tissue sampling. Blood samples were collected into 10 mL tubes and then centrifuged at 3,000 × *g* for 10 min at 4 °C to recover serum samples. The liver, spleen, and kidneys were promptly collected after exsanguination. Liver samples were harvested and rinsed several times with ice-cold phosphate-buffered saline and then immediately snap-frozen in liquid nitrogen and stored at 80 °C for RNA extraction. The liver, spleen, and kidneys were weighed immediately after collection for the subsequent calculation of organ indexes.

### 2.5 Serum parameters

Serum biochemical parameters, including blood urea nitrogen (BUN), triglyceride (TG), Low-Density Lipoprotein Cholesterol (LDLC), high-density lipoprotein cholesterol (HDL-C), total cholesterol (TC) were measured using an automated biochemistry analyzer (Synchron CX Pro, Beckman Coulter, Fullerton, CA, United States) and the commercial kit (Aokai (Suzhou) Biotechnology Co., Ltd., Suzhou, China).

### 2.6 Measurement of antioxidant parameters in liver

The activities of antioxidant-related enzymes in liver homogenate including total antioxidative capacity (T-AOC), superoxide dismutase (SOD), catalase (CAT), and the content of malondialdehyde (MDA) were determinated by kits purchased from Nanjing Jiancheng Bioengineering Institute (Nanjing, China).

### 2.7 Quantitative real-time polymerase chain reaction (RT-qPCR)

According to our previous study (3), total RNA was isolated from the liver tissue, and the concentration and purity of RNA were determined by NanoDrop Oneᶜ (Thermo Fisher Scientific, Waltham, MA, USA). According to the instructions of the Primer-script TM RT Reagent Kit with gDNA Eraser (TaKaRa, Dalian, China; Code No. RR047A), gDNA was removed and reverse-transcribed and the reverse-transcribed cDNA was stored in a refrigerator at −20 °C for later use. According to the manufacturer’s protocol, the RT-qPCR was performed on a Roche LightCyclerfi 480 instrument II (Roche, Basel, Switzerland) with 10 μL total volume reaction, consisting of 5 μL 2X SYBRR Green Pro Taq HS Premix II, 2 μL cDNA template, 0.4 μL each of the forward and reverse primers, and 2.2 μL RNase free water. Relative gene expression levels were analyzed using the 2^(-ΔΔCt)^ method and normalization with *β*-actin as a housekeeping gene. The primers required were shown in Table 2.

**Table 2:** Primer sequences of quantitative real-time PCR.

Gene name	Primer sequences (5′–3′)	Size	Accession no.
*β-actin*	F: TCTGGCACCACACCTTCT R: TGATCTGGGTCATCTTCTCAC	114	XM_021086047.1
*PC*	F: CTAGGGGTGGGTCAGGGTCAA R: TCCTGCTTCATGCCTCACCTT	3,899	NC_010444.4
*PEPCK1*	F: TCTGGTGTACGAGGCTCTCA R: CCTCCTTGGAGACTCACCCT	106	NC_010459.5
*PEPCK2*	F: GCTCCGAGCTTACCAGTTGT R: AACACCTGGCCCATTACCTC	295	NC_010449.5
*G6PC*	F: CCTTCAAGACAACTTCGCCC R: CCAGCCACTTTCTCTTCTGGA	296	NC_010454.4
*SREBP1*	F: CTGTGTGACCTGCTCCTTGT R: CTCATGGAGGAACACCTGGG	289	NC_010454.4
*SCD*	F: CCCGAGTGTCAAGTGGCTTA R: TGATGATGCCGAAATAGCAG	122	NC_010456.5
*CPT1α*	F: TGAATGACGGAACGAGTGGG R: CTCCAACCTAAACGGTGGCT	140	NC_010444.4
*PPARα*	F: GCATTTAGAGGCGTGGCATC R: TCTCAGTTGGGGAGCCTCTT	1,384	NC_010447.5
*FATP1*	F: CATAGCAGGACCAGAGACGC R: ACAAGCCCATTCTCACGCAT	190	NC_010444.4
*NQO1*	F: GCAGTGGCTCCATGTACTCT R: CACCTCAGGTCTGTTTCCAG	529	NC_010448.4
*CAT*	F: GCCGGTGGTCAGGATATCAG R: ACGAGGTACCCTCTGACCTT	105	NC_010444.4
*NRF2*	F: CTGGCTCTTTCTGGGGTTCT R: GTGGGGATGGTAGCTTGGAAA	422	NC_010457.5
*MnSOD*	F: CTTGCAGATTGCCGCTTGTT R: CTCGTCTTCCTCACCTCACG	2046	NC_010443.5
*Cu/ZnSOD*	F: TCCTGGTTTCTAAAGGTCCCC R: AAGACTGCGTCTGTTTCCGT	3,960	NC_010455.5
*HO-1*	F: CTGGACTCTTCTGTTCCCGT R: GACCGGCCACATTTCAACAC	128	NW_018084968.1
*GPX1*	F: CAACCCTTGGGAAGAGTGCC R: AGTTTGGACATCAGGTGCGT	533	NC_010455.5
*GPX4*	F: GTTAGGACAGGGCTTCGACC R: CCCGGTTTTCATCCCTTTGG	250	NC_010444.4

### 2.8 Fatty acids analysis in liver of piglets

Based on our previously published method (13), the fatty acid methyl esters (FAMEs) were prepared from liver samples. Briefly, total lipids were extracted with a chloroform-methanol mixture (2:1, v/v), followed by transesterification using 14% boron trifluoride-methanol to generate FAMEs. The FAMEs were then analyzed using an Agilent 6,890 N gas chromatograph (Agilent Technologies, Santa Clara, CA, USA) equipped with a flame ionization detector and a fused-silica capillary column. Individual fatty acids were identified by comparing their retention times with those of known FAME standards, and their contents were quantified based on peak area, expressed as a percentage of total fatty acids.

### 2.9 Statistical analysis

The original data were preliminarily sorted out by Excel 2010, and the independent sample *t*-test was carried out for the data by SPSS 19.0. Data were expressed as least squares means ± standard error of mean (SEM). The values were considered to be an extremely significant difference when *p* < 0.01, a significant difference when *p* < 0.05 and a tendency when 0.05 < *p* < 0.10.

## 3 Result

### 3.1 Growth performance

As previously reported in a study focused on growth performance from the same experimental cohort dietary LZ supplementation significantly improved the average daily gain in weaned piglets (14).

### 3.2 Organ indexs

Compared with CON, LZ significantly augmented the liver of weaned piglets (*p* < 0.05), but had no significant effect on spleen index and kidney index (*p* > 0.10) (Table 3).

**Table 3:** Effect of LZ on organ indexes of weaned piglets.^1^

Item (%)	CON	LZ	*p*-value
Liver index	0.045 ± 0.0030^b^	0.053 ± 0.0030^a^	0.008
Kidney index	0.005 ± 0.0007	0.004 ± 0.0007	0.235
Spleen index	0.005 ± 0.0004	0.005 ± 0.0004	0.309

CON = control group; LZ = lysozyme group. ^a, b^Within a row, means without a common superscript differ significantly (*P* < 0.05). ^1^Each value represents the mean ± SEM of 6 replicates (*n* = 6).

### 3.3 Serum biochemical parameters

Table 4 showed the serum biochemical indices of weaned piglets. In this study, LZ significantly increased (*p* < 0.05) the concentration of TG, TC, HDL-C and LDLC in serum compared to CON group. However, no significant differences were found in serum BUN indices between the LZ and CON group (*p* > 0.10).

**Table 4:** Effect of LZ on serum biochemistry of weaned piglets.^1^

Item	CON	LZ	*P*-value
BUN, mmol/L	3.75 ± 0.540	3.92 ± 0.360	0.790
TG, mmol/L	0.75 ± 0.110^b^	1.20 ± 0.040^a^	0.004
TC, mmol/L	1.97 ± 0.100^b^	2.51 ± 0.130^a^	0.008
LDLC, mmo/L	1.24 ± 0.080^b^	1.61 ± 0.120^a^	0.027
HDL-C, mmol/L	0.79 ± 0.080^b^	0.99 ± 0.080^a^	0.030

CON = control group; LZ = lysozyme group. ^a, b^Within a row, means without a common superscript differ significantly (*P* < 0.05). ^1^Each value represents the mean ± SEM of 6 replicates (*n* = 6).

### 3.4 Liver antioxidant content

**Figure 1:**
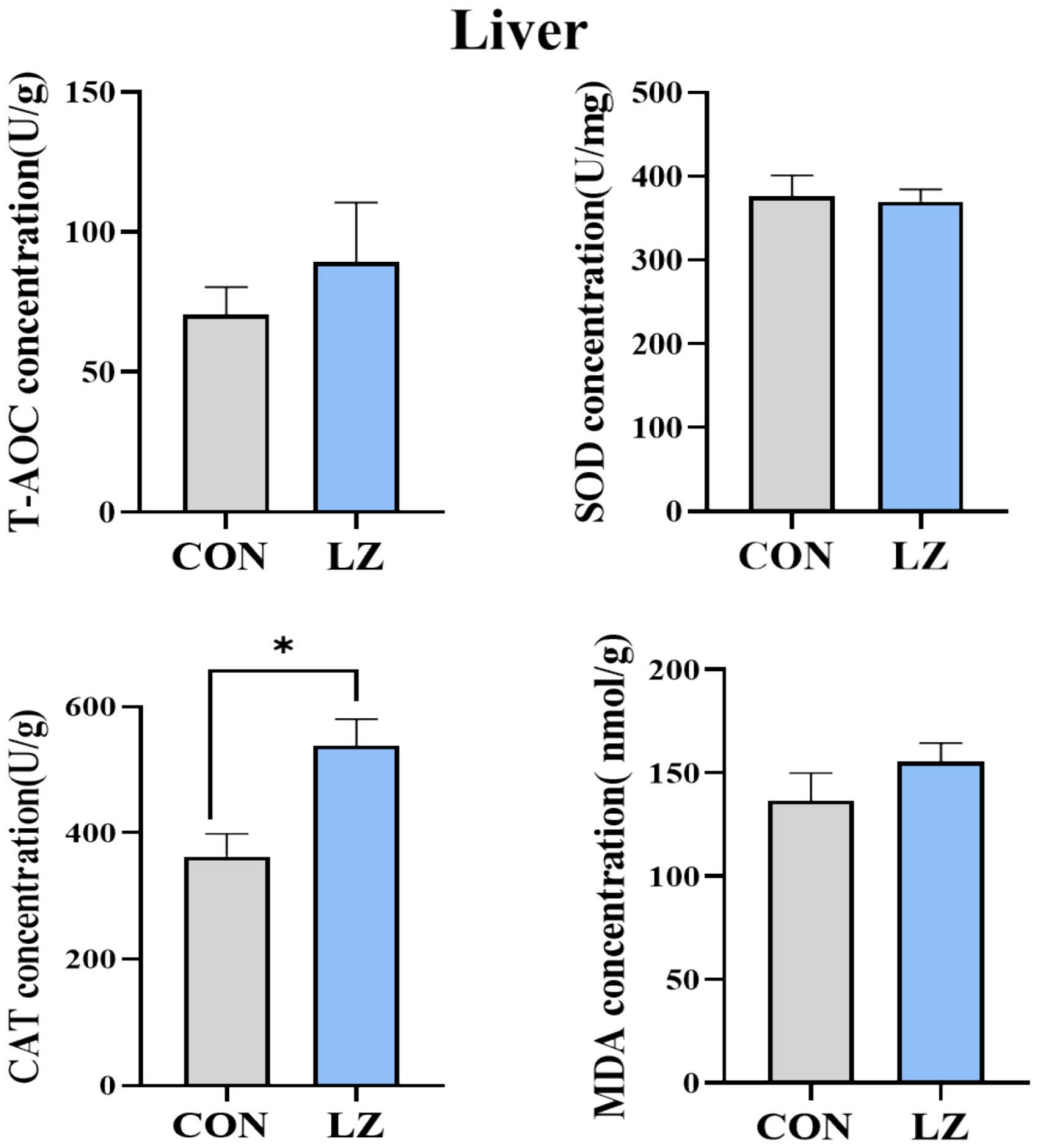
Effects of LZ supplementation on hepatic antioxidant content in weaned piglets. Malonaldehyde. Values are presented as mean ± SEM; * and **indicate a statistically significant difference by *t*-test at *p* < 0.05 and *p* < 0.01. CON = control group; LZ = lysozyme group.

As shown in Figure 1, LZ supplementation affected the liver antioxidant parameters of weaned piglets. The liver CAT activities were significantly increased (*p* < 0.05) in the LZ group when compared with the CON group. There was no significant difference in serum T-AOC, SOD, MDA between the 2 groups.

### 3.5 Antioxidant enzyme gene expression in liver

**Figure 2:**
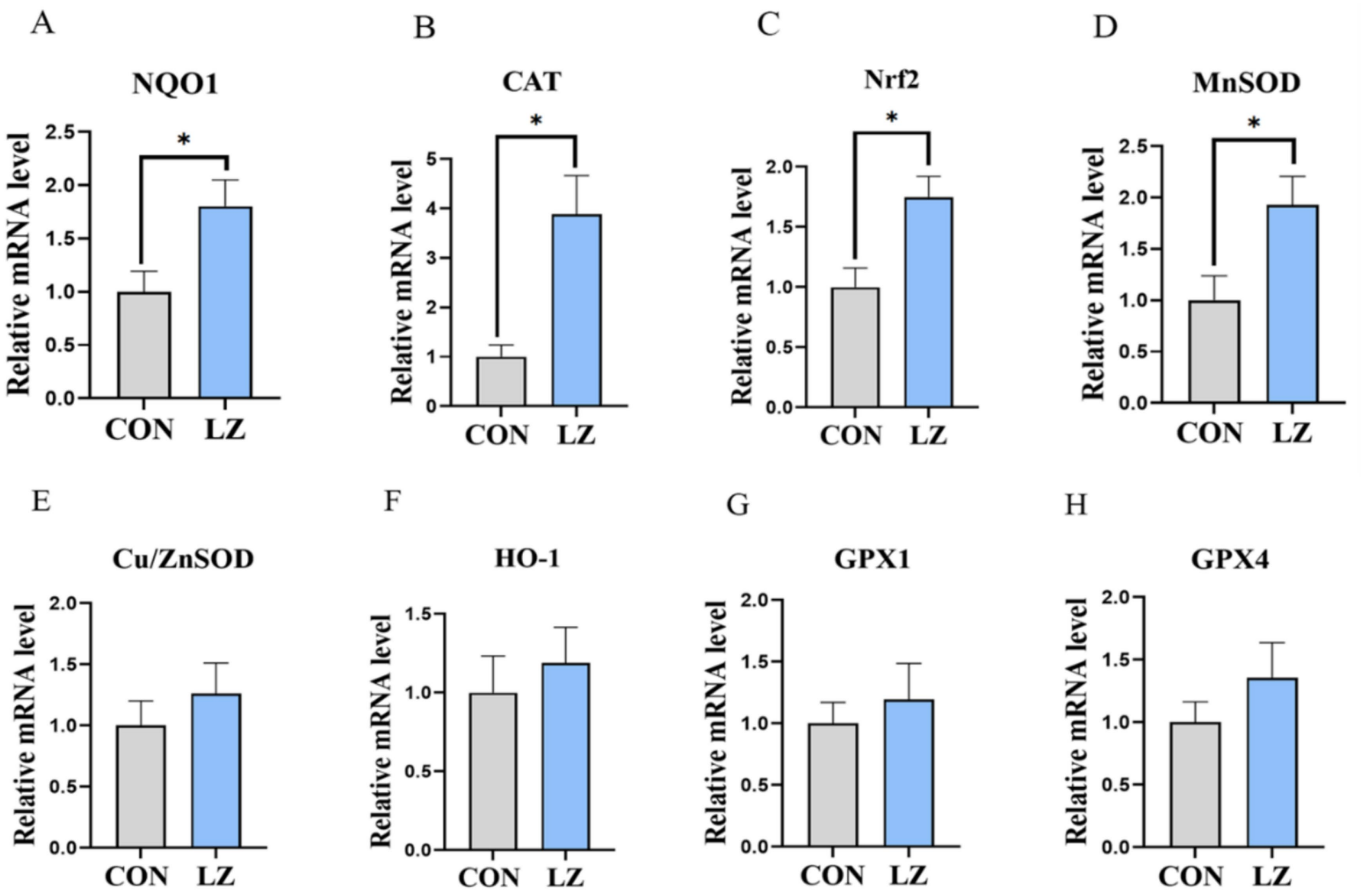
**(A–H)** Effects of LZ supplementation on hepatic antioxidant activity in weaned piglets. Values are presented as mean ± SEM; * and **indicate a statistically significant difference by *t-*test at *p* < 0.05 and *p* < 0.01. CON = control group; LZ = lysozyme group.

We also evaluated the effects of dietary LZ supplementation on antioxidant capacity of weaned piglets (Figure 2). The results exhibited that the mRNA expression of NADH quinone oxidoreductase 1 (*NQO1*), nuclear factor E2-related factor 2 (*Nrf2*), Mn superoxide dismutas (*MnSOD*), and catalase (*CAT*) were significantly up-regulated (*p* < 0.05) of the LZ group compared with the CON group.

### 3.6 Liver fatty acid profile

**Figure 3:**
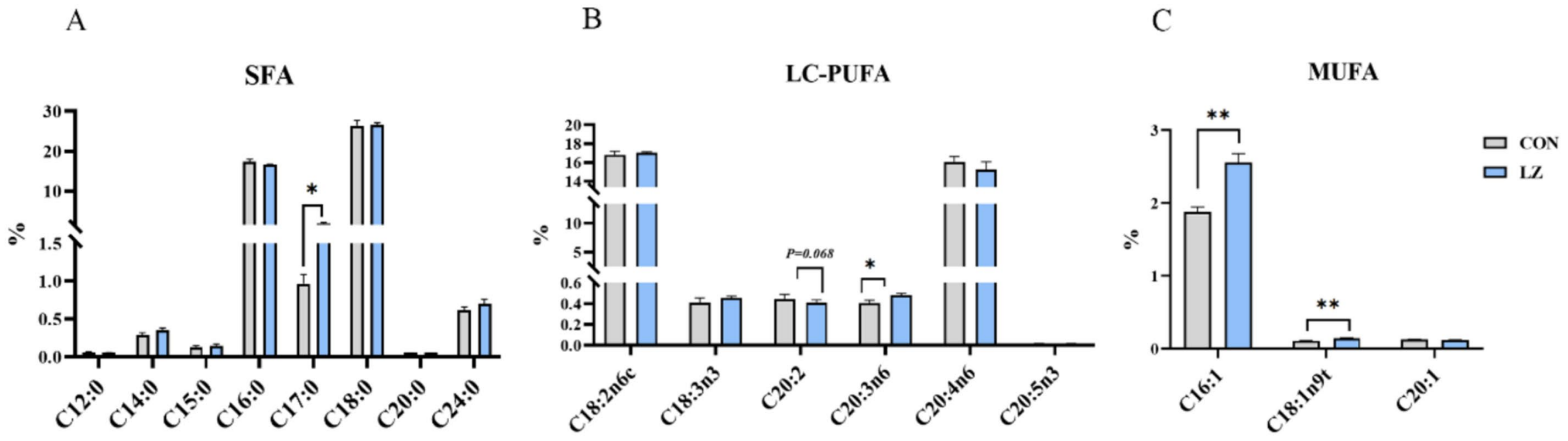
**(A–C)** Effects of LZ supplementation on hepatic fatty acid profile in weaned piglets. SFA, saturated fatty acid (including C12:0, C14:0, C16:0, C15:0, C17:0, C18:0, C20:0, and C24:0); MUFA, monounsaturated fatty acids (including C16:1, C18:1n9t, and C20:1); LC-PUFA, polyunsaturated fatty acids (including C18:2n6c, C18:3n3, C20:2, C20:3n6, C20:4n6, and C22:5n3); Values are presented as mean ± SEM; * and **indicate a statistically significant difference by t-test at *p* < 0.05 and *p* < 0.01. CON = control group; LZ = lysozyme group.

The liver fatty acid profiles of the weaned piglets are shown in Figure 3. Interestingly, it was found that dietary LZ supplementation significantly increased the content of C16:1 fatty acid (*p* < 0.01), C17:0 fatty acid (*p* < 0.05), C20:3n6 fatty acid (*p* < 0.01) and C18:1n9t (*p* < 0.05) fatty acid in liver. Besides, the results showed that the C20:2 (*p* = 0.068) content in the liver of weaned piglets from the LZ group tended to decrease.

### 3.7 Hepatic lipid metabolism-related gene expression

**Figure 4:**
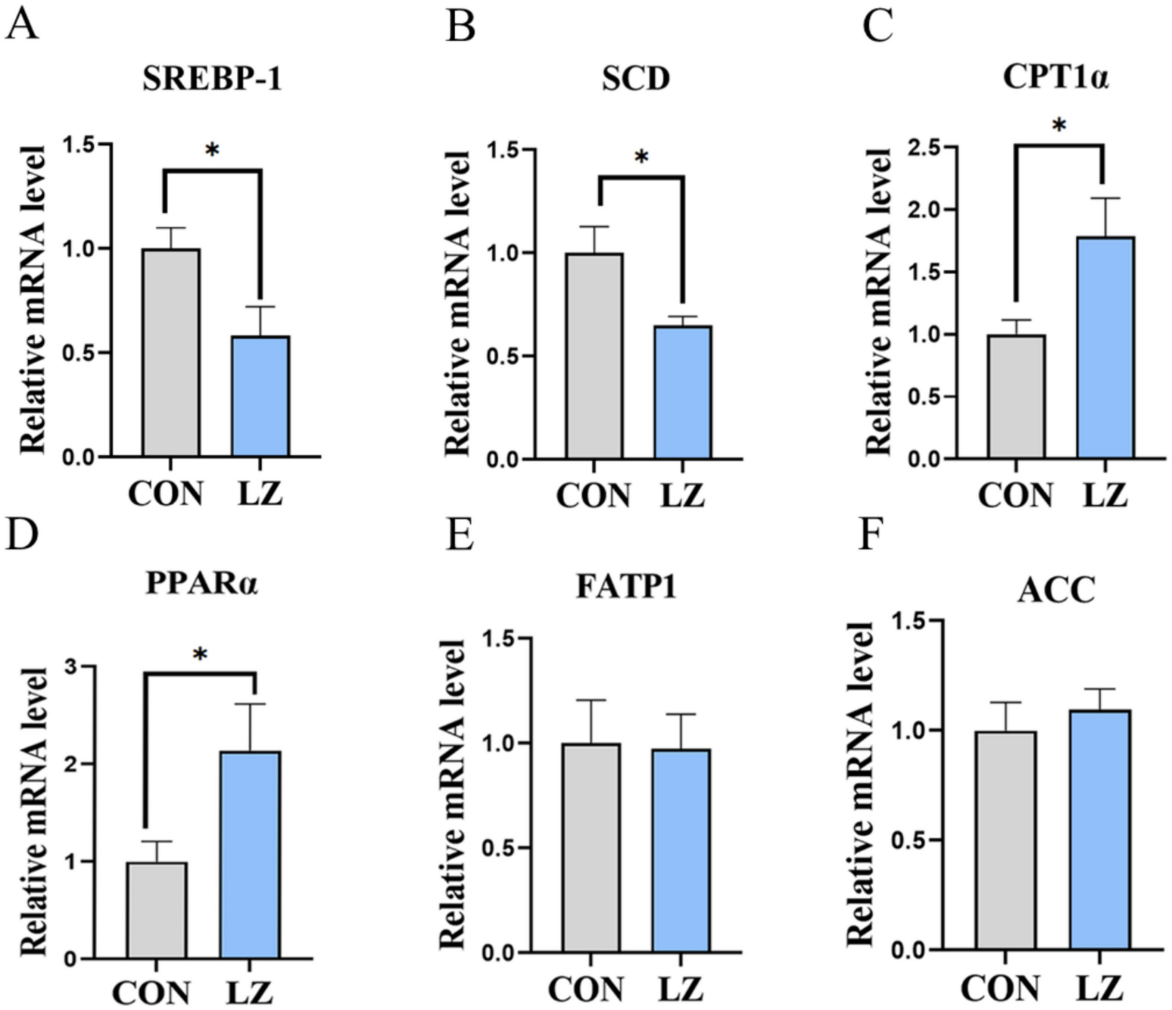
**(A–F)** Effects of LZ supplementation on hepatic lipid metabolism in weaned piglets. Values are presented as mean ± SEM; * and **indicate a statistically significant difference by *t-*test at *p* < 0.05 and *p* < 0.01. CON = control group; LZ = lysozyme group.

To evaluate the effects of LZ supplementation on lipid synthesis, catabolism, and transport of weaned piglets, we measured the related gene expression in the liver (Figure 4). The results showed that the mRNA expression of carnitine palmitoyl transferase 1 *α* (*CPT1α*) and peroxisome proliferator activated receptors (*PPARα*) were significantly increased (*p* < 0.05), and stearoyl-CoA desaturase (*SCD*) and sterol regulatory element-binding protein 1 (*SREBP1*)were significantly down-regulated (*p* < 0.05) in the liver in the LZ group compared with the CON group.

### 3.8 Gene expression of liver gluconeogenesis

**Figure 5:**
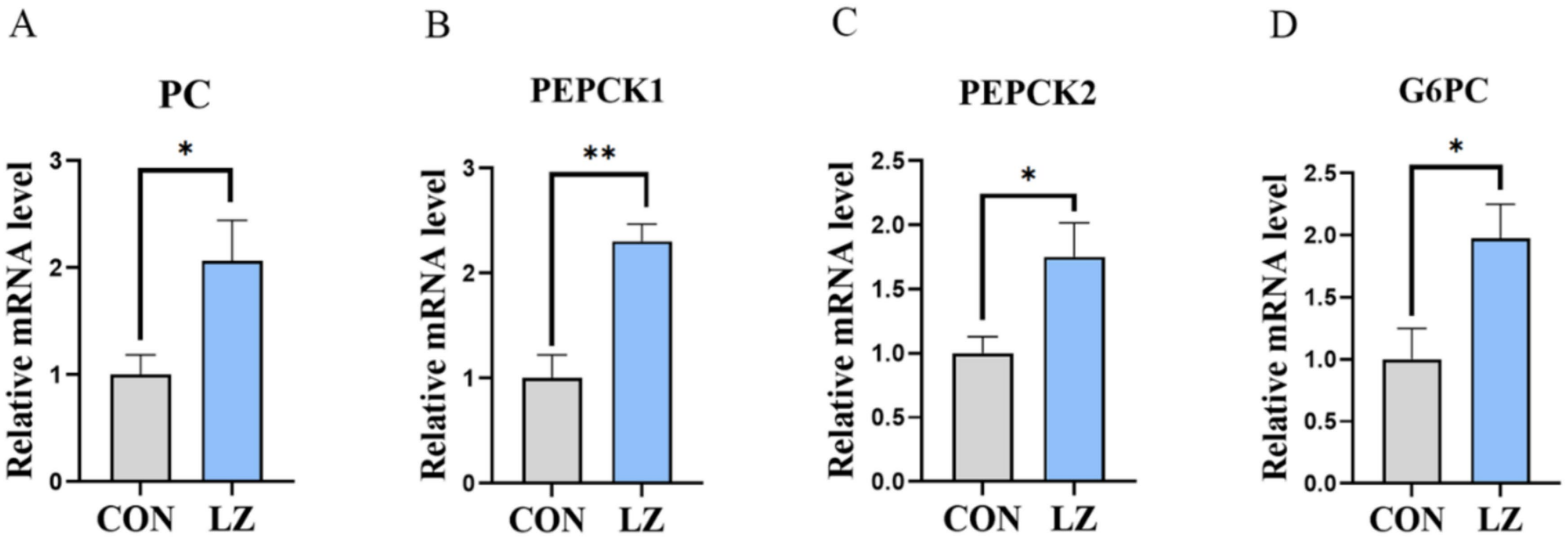
**(A–D)** Effects of LZ supplementation on hepatic glucose metabolism in weaned piglets. Values are presented as mean ± SEM; * and **indicate a statistically significant difference by *t-test* at *p* < 0.05 and *p* < 0.01. CON = control group; LZ = lysozyme group.

Relative mRNA expression of genes related to glucose metabolism in the liver (Figure 5) showed that dietary LZ could significantly increase expression of phosphoenolpyruvate carboxykinase (*PEPCK*), pyruvate carboxylase (*PC*) and Glucose-6-phosphate dehydrogenase (*G6PC*).

## 4 Discussion

The liver participates in complex physiological processes, including lipid metabolism, glucose homeostasis, and oxidative stress, which play crucial roles in maintaining normal energy metabolism (4, 15). However, weaning stress induces hepatic oxidative stress and oxidative damage, severely impairing liver function in piglets. LZ is a safe, non-toxic biological enzyme with strong antioxidant properties (16–18). LZ contains a high proportion of hydrophobic and positively charged amino acids, whose antioxidant activities are markedly enhanced upon hydrolysis. Moreover, LZ can combine with other substances to further enhance antioxidant capacity (19, 20). Previous research demonstrated that dietary LZ supplementation significantly increased intestinal levels of SOD1 and GSH-Px in broiler chickens, indicating a beneficial effect on antioxidative capacity (11). Although few studies have reported the direct effects of LZ on glycolipid metabolism in weaned piglets, some studies suggested that LZ may indirectly influence glycolipid metabolism by modulating other biological functions (21). Thus, this study aimed to investigate the effects of LZ supplementation on hepatic glucolipid metabolism and antioxidant function in weaned piglets.

To some extent, the organ index can reflect organ function (22). The liver is the most metabolically active organ in the body and the largest digestive gland, responsible for glycogen storage, bile secretion, and synthesis of secretory proteins (23). Our results showed that LZ significantly increased the liver index in weaned piglets, indicating that LZ promoted hepatic development. Blood lipid concentrations reflect the status of lipid metabolism in the body (24). In this study, dietary LZ significantly increased serum levels of TC, TG, and LDLC compared to the control group. Cholesterol (CHOL) is released from the liver into the bloodstream, carried by LDL, and transported throughout the body to provide metabolic energy and support other vital activities (19, 20). Elevated LDLC and cholesterol levels may indicate improved nutrient absorption in rapidly growing piglets (25, 26). Collectively, our results suggest that LZ can enhance hepatic function in weaned piglets.

Under normal physiological conditions, animals maintain a state of dynamic redox balance. However, stressors such as weaning can stimulate excessive production of reactive oxygen species (ROS), disrupting this redox equilibrium (27). Research has demonstrated that the liver of weaned piglets contains elevated levels of free radicals and significant lipid peroxidation (28). In this study, dietary LZ supplementation significantly increased catalase (CAT) activity in the liver of weaned piglets. To further elucidate the antioxidant mechanisms of LZ supplementation in weaned piglets, we examined gene expression levels. The Nrf2 signaling pathway constitutes a crucial defense mechanism against oxidative damage, promoting the transcription of multiple downstream protective proteins (29, 30). Our results revealed that LZ significantly upregulated the mRNA expression of the transcription factor Nrf2, a key regulator of antioxidant responses. The subsequent upregulation of its downstream target genes, including NQO1 and MnSOD, confirmed the activation of this cytoprotective pathway. Importantly, increased CAT mRNA expression correlated with significantly elevated catalase enzyme activity. This coordinated enhancement at both transcriptional and functional levels suggests that LZ fortifies the hepatic antioxidant defense system by activating the Nrf2 pathway, thus improving the ability of piglets to counteract oxidative stress (31).

A previous study indicated that lysosomal membrane proteins (LMPs) are closely associated with glucose and lipid metabolism (21). However, whether LZ affects glucolipid metabolism in weaned pigs and its underlying mechanisms remain unclear. Medium-chain fatty acids (MCFAs) and long-chain fatty acids (LCFAs) are efficiently absorbed and metabolized by piglets, providing immediate energy (19, 20). We evaluated hepatic fatty acid profiles in weaned piglets to determine the effects of LZ on fatty acid composition. We observed that LZ significantly increased the content of palmitoleic acid (C16:1), C17:0, C20:3n6, and C18:1n9t. As previously reported, increased C17:0 content is associated with ferritin, inflammation, and elevated glucose, triglyceride, and insulin levels (19, 20). As an unsaturated fatty acid, C16:1 participates in energy metabolism, inflammatory response regulation, cell signaling, antioxidant activity, and cell membrane structure and maintenance (32). C18:1n9t is a highly unsaturated fatty acid with significant potential in lipid metabolism regulation, cardiovascular protection, and anti-cancer activity (33). In addition, C20:2 content tended to decrease. This reduction in C20:0 proportion is associated with ceramide synthesis (19, 20).

The key processes of lipid metabolism include lipogenesis, lipolysis, and fatty acid oxidation, with the liver being the primary organ for fatty acid synthesis and utilization (34). Therefore, the mRNA expression levels of lipid metabolism-related genes were evaluated to further investigate the effects of LZ on lipid metabolism. Carnitine palmitoyltransferase 1 alpha (CPT1α) is a rate-limiting enzyme in fatty acid *β*-oxidation, responsible for converting long-chain fatty acids into fatty acyl-coenzyme A, which subsequently enters mitochondria for β-oxidation to generate ATP and energy (35, 36). Peroxisome proliferator-activated receptor alpha (PPARα) is a nuclear transcription factor involved in regulating intracellular fatty acid uptake and triglyceride catabolism, as well as controlling *CPT1α* expression (37). Our results demonstrated that hepatic mRNA expression of *CPT1α* and *PPARα* was significantly upregulated in the LZ group compared with the CON group, suggesting that LZ promotes lipid catabolism in weaned piglets by regulating *CPT1α* expression. Furthermore, dietary LZ supplementation significantly increased the hepatic mRNA expression of stearoyl-CoA desaturase (SCD) and sterol regulatory element-binding protein 1 (*SREBP-1*). SCD, primarily expressed in adipose tissue, is crucial for synthesizing monounsaturated fatty acids and determining the composition of phospholipids and triglycerides in cellular membranes (38). SREBP-1 plays an essential role in cholesterol biosynthesis and regulates lipid synthesis by activating the expression of lipid metabolism-related genes (39, 40). Thus, dietary LZ supplementation could enhance hepatic lipid metabolism in piglets.

The beneficial effects of LZ supplementation on hepatic function and systemic lipid metabolism are closely linked to its antioxidant properties. Activation of the Nrf2 signaling pathway enhanced the hepatic antioxidant defense system, as evidenced by increased CAT activity and upregulated expression of NQO1 and MnSOD. This improvement in antioxidant capacity likely established favorable conditions for lipid metabolic regulation. Given that oxidative stress impairs mitochondrial function and promotes lipotoxicity, LZ-mediated alleviation of oxidative stress may have contributed to the upregulation of PPARα and its downstream target CPT1α, which are critical regulators of fatty acid *β*-oxidation. Simultaneously, the downregulation of SREBP-1 and SCD suggests suppressed lipogenesis. Collectively, this metabolic shift from lipid accumulation toward oxidation and utilization provides a plausible explanation for the improved serum lipid profile and supports the conclusion that LZ enhances hepatic function. These findings demonstrate that LZ, by activating Nrf2-mediated antioxidant responses, promotes lipid metabolic reprogramming that ultimately benefits liver health.

In the case of long-term stress, animals regulate glucose homeostasis to meet energy demands (41). After birth, piglets rapidly deplete glycogen stores, making hepatic gluconeogenesis, the primary energy source for weaned piglets, extremely important for growth (42, 43). Gluconeogenesis is directly controlled by rate-limiting enzymes, including PEPCK, G6PC, and PC (44, 45). Although many tissues express gluconeogenic enzymes, only the liver and kidney express G6PC, which enables glucose release into the circulation and contributes to the systemic glucose pool. The gluconeogenic pathway involves both mitochondrial and cytosolic enzymes, and two forms of PEPCK have been identified in the liver of most mammalian species: mitochondrial PEPCK-2 and cytosolic PEPCK-1 (46). In the present study, analysis of mRNA expression levels of gluconeogenesis-related genes showed that LZ supplementation significantly increased the expression of key rate-limiting enzymes, including *PC*, *G6PC*, *PEPCK1*, and *PEPCK2*. Therefore, dietary LZ may enhance hepatic gluconeogenesis and help maintain glucose metabolic balance in weaned piglets by upregulating essential gluconeogenic enzymes.

## 5 Conclusion

In conclusion, we demonstrated that LZ could promote liver function such as improve liver lipid metabolism, enhance liver gluconeogenesis to maintain the balance of glucose metabolism in weaned piglets. Furthermore, we found LZ improve the antioxidant ability of weaned piglets by regulating Nrf2 signaling pathway and increasing antioxidant enzyme activities. This study provided an important theoretical basis for LZ to become a feed additive with broad application prospects in pig production.

## Data Availability

The raw data supporting the conclusions of this article will be made available by the authors, without undue reservation.

## Glossary

LZ: Lysozyme
BUN: Blood urea nitrogen
TG: Triglyceride
LDLC: Low-Density Lipoprotein Cholesterol
HDL: High-Density Lipoprotein Cholesterol
TC: Total Cholesterol
*β*-actin: β-non muscle actin
PC: Pyruvate carboxylase
PEPCK1: Phosphoenolpyruvate carboxykinase 1
PEPCK2: Phosphoenolpyruvate carboxykinase 2
G6PC: Glucose 6-phosphatase
SREBP1: Sterol regulatory element-binding protein-1
SCD: Stearoyl-CoA desaturase
CPT1*α*: Carnitine palmitoyl ltransferase1 α
PPARα: Peroxisomal Proliferator-activated Receptor α
FATP1: Fatty acid transport protein 1
ACC: Acetyl-CoA Carboxylase
NQO1: NADH quinone oxidoreductase 1
CAT: Catalase
Nrf2: Nuclear factor E2-related factor 2
MnSOD: Mn Superoxide dismutase
Cu/ZnSOD: Cu/Zn-Superoxide Dismutase
HO-1: Heme Oxygenase-1
GPX1: Glutathione peroxidase 1
GPX4: Glutathione peroxidase 4
T-AOC: Total antioxidant capacity
SOD: Superoxide dismutase
MDA: Malonaldehyde
